# Do Measures of Real-World Physical Behavior Provide Insights Into the Well-Being and Physical Function of Cancer Survivors? Cross-Sectional Analysis

**DOI:** 10.2196/53180

**Published:** 2024-07-15

**Authors:** Shelby L Bachman, Emma Gomes, Suvekshya Aryal, David Cella, Ieuan Clay, Kate Lyden, Heather J Leach

**Affiliations:** 1 VivoSense, Inc Newport Coast, CA United States; 2 Department of Health and Exercise Science Colorado State University Fort Collins, CO United States; 3 Feinberg School of Medicine Northwestern University Chicago, IL United States

**Keywords:** accelerometer, cancer survivorship, cancer survivors, digital health technology, health-related quality of life, physical behavior, physical function

## Abstract

**Background:**

As the number of cancer survivors increases, maintaining health-related quality of life in cancer survivorship is a priority. This necessitates accurate and reliable methods to assess how cancer survivors are feeling and functioning. Real-world digital measures derived from wearable sensors offer potential for monitoring well-being and physical function in cancer survivorship, but questions surrounding the clinical utility of these measures remain to be answered.

**Objective:**

In this secondary analysis, we used 2 existing data sets to examine how measures of real-world physical behavior, captured with a wearable accelerometer, were related to aerobic fitness and self-reported well-being and physical function in a sample of individuals who had completed cancer treatment.

**Methods:**

Overall, 86 disease-free cancer survivors aged 21-85 years completed self-report assessments of well-being and physical function, as well as a submaximal exercise test that was used to estimate their aerobic fitness, quantified as predicted submaximal oxygen uptake (VO_2_). A thigh-worn accelerometer was used to monitor participants’ real-world physical behavior for 7 days. Accelerometry data were used to calculate average values of the following measures of physical behavior: sedentary time, step counts, time in light and moderate to vigorous physical activity, time and weighted median cadence in stepping bouts over 1 minute, and peak 30-second cadence.

**Results:**

Spearman correlation analyses indicated that 6 (86%) of the 7 accelerometry-derived measures of real-world physical behavior were not significantly correlated with Functional Assessment of Cancer Therapy-General total well-being or linked Patient-Reported Outcomes Measurement Information System-Physical Function scores (*P*s≥.08). In contrast, all but one of the physical behavior measures were significantly correlated with submaximal VO_2_ (*P*s≤.03). Comparing these associations using likelihood ratio tests, we found that step counts, time in stepping bouts over 1 minute, and time in moderate to vigorous activity were more strongly associated with submaximal VO_2_ than with self-reported well-being or physical function (*P*s≤.03). In contrast, cadence in stepping bouts over 1 minute and peak 30-second cadence were not more associated with submaximal VO_2_ than with the self-reported measures (*P*s≥.08).

**Conclusions:**

In a sample of disease-free cancer survivors, we found that several measures of real-world physical behavior were more associated with aerobic fitness than with self-reported well-being and physical function. These results highlight the possibility that in individuals who have completed cancer treatment, measures of real-world physical behavior may provide additional information compared with self-reported and performance measures. To advance the appropriate use of digital measures in oncology clinical research, further research evaluating the clinical utility of real-world physical behavior over time in large, representative samples of cancer survivors is warranted.

**Trial Registration:**

ClinicalTrials.gov NCT03781154; https://clinicaltrials.gov/ct2/show/NCT03781154

## Introduction

### Background

As a result of progress in early cancer detection and the development of effective anticancer therapies, the number of individuals who have survived cancer is increasing. As of 2022, >18 million individuals in the United States were living with a history of cancer [1]. In the future, this number is projected to increase as the aging population grows and cancer screening, treatment, and survivorship care continue to advance [2,3]. Although increases in cancer survivorship are cause for optimism, clinicians and regulators alike are increasingly interested in ensuring that increases in cancer survival rates translate to additional years of good quality life [4,5].

Cancer and its treatments have major impacts on health-related quality of life [6]. These effects persist long into survivorship, with more than one-third of cancer survivors reporting that symptoms persist after treatment ends [7-9]. Across studies, individuals off cancer treatment, henceforth referred to as *cancer survivors*, report reductions in physical performance, fatigue, sleep problems, mood disturbances, and pain as long-term symptoms, even years after being disease free [9-11]. The impacts of cancer and its treatments are also associated with poorer outcomes and survival in the long term. For instance, individuals who experience a greater health burden from cancer symptoms are at an elevated risk of developing chronic comorbidities [12]. Furthermore, among adults with a history of cancer, both depression [13,14] and reduced physical function [15-18] are associated with an increased risk of mortality after controlling for confounding variables. At the same time, there is accumulating evidence that in cancer survivorship, health-promoting behaviors have positive impacts; for instance, exercise interventions have been demonstrated to improve health-related quality of life, objectively assessed physical function, and aerobic fitness in cancer survivors [19-21].

Therefore, understanding and considering the long-term impacts of anticancer therapies on health-related quality of life should be an integral component of assessing risk-benefit profiles during both regulatory and medical decision-making. This necessitates methods to accurately and reliably capture features of health-related quality of life that are important to cancer survivors. Established methods to assess these constructs in oncology clinical research include patient-reported assessments of global and domain-specific well-being, clinician-reported assessments of functional capacity, and performance assessments that capture physical performance capacity [22,23]. Collectively, these assessments have a range of limitations: patient-reported outcome assessments are burdensome and prone to floor and ceiling effects [24], clinician-reported outcome assessments exhibit limited interobserver reliability [25,26], and performance outcome assessments do not reflect many of the day-to-day functional challenges experienced by those with a history of cancer. Together, these limitations raise the question of how to best capture how cancer survivors are feeling and functioning in their real-world environments.

In the midst of a digital transformation in medicine, there is a growing interest in digital health technologies as measurement tools in oncology clinical care and research [27,28]. In particular, wearable sensors such as accelerometers have the potential to address some of the limitations of the established assessments of health-related quality of life in oncology [29]. These technologies can capture aspects of everyday physical behavior remotely (in individuals’ lived environment), passively (as individuals go about their daily lives), and continuously (with high granularity) [28,30]. These devices can furthermore capture many domains of physical behavior, including aspects of gait, mobility, posture, physical activity, and sedentary behavior [31-33]. Alongside established outcome assessments, these measures may provide rich insights into the real-world well-being and physical function in cancer survivorship [34-36].

The use of wearable sensors as monitoring tools in oncology clinical research is on the rise [37], but despite their potential for capturing how individuals feel and function in their real-world environments, these tools have not been widely adopted for assessing treatment efficacy or monitoring in cancer clinical research. Furthermore, across trials that have deployed wearable sensors, there is little standardization regarding which outcome measures are included, as well as the definitions of those measures [37]. Together with this lack of standardization, a potential reason for the limited adoption of digital measures derived from wearable sensors is that there is limited clinical validation evidence linking specific digital measures of real-world physical behavior to gold-standard outcome measures commonly used in oncology clinical research (ie, patient-reported, clinician-reported, and performance outcomes) [29].

### Objectives

In this secondary analysis, we aimed to gain insight into how various digital measures of real-world physical behavior, captured with wearable sensors, can provide an additional understanding of health-related quality of life following cancer treatment. To do so, we leveraged data from 2 previous studies of individuals who had completed cancer treatment to test whether an array of digital measures of real-world physical behavior, measured with a wearable accelerometer over a 1-week period, were related to self-reported and performance measures of physical function. First, we examined associations between real-world physical behavior and self-reported well-being and physical function. Next, we examined how real-world physical behavior was related to aerobic fitness, captured with a submaximal exercise test performed in the clinic. Finally, we compared these patterns of associations to determine whether real-world physical behavior was more closely related to self-reported well-being and physical function or to aerobic fitness.

## Methods

### Overview

Data were collected as part of 2 studies. Study 1 was a cross-sectional study conducted at Colorado State University between January 2020 and June 2021 and aimed to examine how reallocating time to physical activity affected body composition and quality of life in individuals who had completed cancer treatment [38,39]. Study 2 was a randomized clinical trial conducted at the University of Colorado Anschutz Medical Campus and Colorado State University and aimed to examine the feasibility and preliminary effects of a videoconference physical activity intervention in individuals who had completed treatment for colorectal cancer [40,41]. For study 2, only data collected at the baseline measurement time point (ie, before the initiation of the intervention) were used. These data were collected between February 2021 and July 2022. For increased statistical power, we combined data from studies 1 and 2.

### Ethical Considerations

#### Study 1

The study protocol was approved by Colorado State University Institutional Review Board (IRB #19-8914H). All participants provided written, informed consent before participation and were compensated US $25 for participation. When providing consent, participants consented to their deidentified data being used for future studies. Data were deidentified before analyses.

#### Study 2

The study protocol was approved by the University of Colorado Institutional Review Board (IRB #18-2436). Informed consent was obtained from all participants. As a part of this process, participants consented to their deidentified data being used for research purposes beyond the primary study aims. Participants were compensated up to US $75 for participation. Data were deidentified before analyses.

### Participants

#### Study 1

Participants in study 1 were recruited from local and regional cancer centers and the Colorado State University Center for Healthy Aging using flyers, presentations, and email postings. Eligible participants were aged >18 years at the time of their cancer diagnosis and within 60 months of treatment completion at the time of study participation.

#### Study 2

Participants in study 2 were recruited from the University of Colorado Cancer Center, survivor support organizations, and community outreach using mailed letters, flyers, and social media platforms. Eligible participants (1) were fluent in English, (2) had access to a computer or phone with internet and a camera, (3) stated willingness to comply with all study procedures and be available for the duration of the study, (4) were male or female individuals aged ≥40 years at the time of diagnosis, (5) had histologically confirmed cancer of the colon or rectum (stages II-IV) if treated with curative intent, completed resection or other surgery 3 to 60 months before enrollment, received chemotherapy and/or radiation therapy within the previous year with at least 1 cycle of intended chemotherapy completed (not necessary to have completed all cycles), and had no plans for additional chemotherapy or radiation therapy. Exclusion criteria were evidence of metastatic disease, existing participation in at least 150 minutes per week of at least moderate intensity physical activity, being pregnant or planning to become pregnant, and known contraindications for exercise.

### Procedure

We aimed to test relationships between participants’ real-world physical behavior, self-reported well-being and physical function, and aerobic fitness; therefore, we focused only on relevant assessments that were included in both studies. These assessments are described in subsequent sections.

#### Assessments of Self-Reported Well-Being and Physical Function

In the laboratory, participants completed a series of questionnaires in which they reported demographic information and information about their cancer diagnosis and types of treatment completed. They also completed the Functional Assessment of Cancer Therapy-General (FACT-G), a 27-item instrument designed to assess health-related quality of life in individuals with cancer along 4 dimensions: physical, functional, emotional, and social well-being [42]. For FACT-G and its subscales, higher scores indicate better well-being.

#### Assessment of Aerobic Fitness

Following the questionnaires, participants completed a submaximal exercise test that involved a modified Balke Treadmill Test. The modified Balke Treadmill protocol consisted of a 3-minute warm-up at a treadmill speed of 2.5 mph. Following the warm-up, participants entered stage 1 of the test at 0% grade and 2.5 mph. Every 3 minutes, participants entered a new stage, increasing the treadmill grade by 2.5% until 70% heart rate reserve was reached or until there was a safety indication to stop the exercise test. Heart rate was collected every minute throughout the protocol.

A measure of aerobic fitness, that is, predicted oxygen uptake (VO_2_) at 70% heart rate reserve, was then calculated according to the following formula (for women [43]; for men [44]), where *T* denotes the test duration (ie, time to reach 70% heart rate reserve):

Predicted submaximal VO_2_ (mL/kg/min) for women = 1.38 (*T*) + 5.22 **(1)**

Predicted submaximal VO_2_ (mL/kg/min) for men = 1.44 (*T*) + 14.99 **(2)**

#### Assessment of Real-World Physical Behavior

At the end of the laboratory visit, participants were instructed that during the subsequent 7-day period, their real-world behavior would be monitored continuously using an activPAL3 activity monitor (PAL Technologies Ltd), worn on the thigh [45]. Using an accelerometer to sense limb position and activity, activPAL can discriminate between the activities of lying, sitting, standing, and stepping and therefore allows for the calculation of time spent in various physical activity categories [46-48]. The sensor identifies reciprocal leg movements as steps, and based on the detected steps, measures including cadence and time in stepping bouts of various durations can be calculated [49].

Participants were each given an activPAL and instructed regarding proper use and wear of the device. Each participant was instructed to wear the device on their thigh for 7 days in their real-world environments. A 7-day monitoring period has been demonstrated to provide sufficient accelerometer data for generating reliable estimates of various measures of real-world physical behavior [50-52]. After the remote monitoring period, participants returned their devices to the laboratory. If their appointment to return the device was >7 days after the beginning of the remote monitoring period, participants were permitted to wear the device longer than 7 days to avoid losing it. All available activPAL data were used for analysis.

### Analysis

#### Linkage of PROMIS-Physical Function Scores

To assess self-reported physical function, we first calculated scores on a 5-item subset of the FACT-G physical well-being subscale. The 5 items in the subset were “I have a lack of energy,” “Because of my physical condition, I have trouble meeting the needs of my family,” “I have pain,” “I feel ill,” and “I am forced to spend time in bed.” This 5-item subset excluded 2 items on the FACT-G physical well-being subscale: “I have nausea” and “I am bothered by the side effects of treatment.” These 5-item subset scores were linked to T scores on a custom subset of the Patient-Reported Outcomes Measurement Information System-Physical Function (PROMIS-PF) calibrated item bank, using an established linkage method [53]. PROMIS-PF is a tool for assessing physical function in oncology clinical research [54,55], for which higher T scores indicate better physical function. We used the linkage procedure described by Kaat et al [53] rather than administering the PROMIS-PF assessment directly.

#### Summarization of Self-Reported and Performance Measures in the Sample

For the purposes of analysis, FACT-G scores, linked PROMIS-PF T scores, and submaximal VO_2_ values were treated as continuous variables. Summary statistics were used to summarize the sample in terms of FACT-G total well-being scores, FACT-G physical well-being subscale scores, scores on the 5-item subset of the FACT-G physical well-being subscale used for linkage to PROMIS-PF, linked PROMIS-PF T scores, and submaximal VO_2_. Ceiling effects, defined as the percentage of the sample achieving the maximum possible score [56], were calculated for each self-reported measure. The skewness and kurtosis of each self-reported measure’s distribution were also calculated.

#### Calculation of Measures of Real-World Physical Behavior

Average daily nonwear time, defined as the time in which participants did not wear the activPAL monitor, was calculated for each participant. A valid day was considered as the one in which a participant wore the monitor for at least 10 hours; only participants with at least 4 valid days during the remote monitoring period were included for analysis [57].

The activPAL proprietary software, PALbatch (version 8.11.1.63; PAL Technologies), was used to access summaries of recorded data and whole recording outcomes from the real-world monitoring period. Measures of interest included average daily time spent sedentary (ie, secondary lying, defined as sitting or lying not classified as primary lying); time in light physical activity; time in moderate to vigorous physical activity; and step count. Average daily time in light and moderate to vigorous intensity activity was calculated using established approaches [47]. In addition, we calculated the average daily time that each participant spent in stepping bouts of ≥1 minute in duration. Finally, we extracted 2 measures of cadence: weighted median cadence in stepping bouts of ≥1 minute across all valid days, as well as the number of steps in any 30-second recording period (“peak 30 s cadence”) across all valid days, a measure that is thought to reflect an individual’s best natural effort [58-60]. Summary statistics were used to characterize the sample in terms of the various measures of real-world physical behavior.

#### Intercorrelations Among Related Measures

As preliminary tests for expected intercorrelations among the self-reported measures and among the measures of real-world physical behavior, we performed Spearman correlation analyses.

#### Associations With Measures of Real-World Physical Behavior

Pairwise Spearman correlation analyses were then used to test for associations between each measure of real-world physical behavior and (1) the self-reported measures and (2) aerobic fitness. These analyses were repeated in a partial Spearman correlation framework to account for the effects of age, sex, BMI, time since diagnosis, and cancer stage at diagnosis on each association. In addition, to test for differences in physical behavior based on the level of self-reported physical function and well-being, we first performed a tertile split of each self-reported measure and a median split of aerobic fitness. A median split instead of a tertile split was performed for aerobic fitness since fewer participants had values of submaximal VO_2_ available compared with the self-reported measures. In cases where scores were equal to a tertile value, they were assigned such that the resulting splits reflecting high, medium, and low scores were approximately equal in size. Then, we used 2-tailed pairwise Welch *t* tests and Mann-Whitney *U* tests to compare the splits in terms of the various measures of real-world physical behavior. Welch *t* tests were used to compare splits in terms of measures that did not exhibit deviations from normality, whereas Mann-Whitney *U* tests were performed to compare splits in terms of measures that exhibited deviations from normality. Deviation from normality was indicated by a statistically significant Shapiro-Walk test result. For each comparison of the splits of self-reported measures, *P* values were adjusted for multiple comparisons using Holm method [61]. For comprehensiveness, we also performed Spearman correlation analyses to test for associations between aerobic fitness and each of the self-reported measures.

#### Comparison of Associations With Measures of Real-World Physical Behavior

A series of likelihood ratio tests was used to determine if the strength of associations with real-world physical behavior differed between the self-reported measures and aerobic fitness. The following steps were performed for each measure of real-world physical behavior. Here, we describe the process for FACT-G total well-being, but the same process was used for FACT-G physical well-being, FACT-G physical well-being 5-item subset, and linked PROMIS-PF T scores:

1. One multiple linear regression model was fit, with all measures (FACT-G total well-being, submaximal VO_2_, age, sex, BMI, time since diagnosis, and cancer stage) regressed onto the measure of real-world physical behavior.
2. A second multiple linear regression model was fit, which was identical to the first, with the exception that the regression coefficients for FACT-G total well-being and submaximal VO_2_ were constrained to equality.
3. A likelihood ratio test was performed to compare the fits of the first (unconstrained) and second (constrained) models; a significant test result indicated that constraining the coefficients to equality led to a significantly poorer model fit.

#### Exploratory Analysis of Associations With Activity Fragmentation

For additional insights into real-world physical behavior, we calculated measures of activity fragmentation, reflecting how participants accumulated their total activity and sedentary time across the days of the remote monitoring period [62]. More fragmented activity patterns have been associated with increased mortality risk, reduced physical function as measured with in-clinic physical performance tests, and fatigability [63,64]. Using a similar approach as mentioned in the *Comparison of Associations With Measures of Real-World Physical Behavior* section, we tested whether the various measures of activity fragmentation were more associated with the self-reported measures or with aerobic fitness (Multimedia Appendix 1 [62,65,66]).

For each analysis comparing regression coefficients, data were restricted to include only those participants with no missing values for the respective measures being compared (ie, the self-reported measure of interest and submaximal VO_2_). In addition, for all linear regression analyses, continuous variables were standardized, and binary variables were coded with a sum contrast coding scheme before analysis. All analyses were performed with R (version 4.1.2; The R Foundation).

## Results

### Overview

For study 1, we screened 101 individuals for participation and enrolled 59 (58.4%) individuals; 2 enrolled participants did not undergo remote monitoring. For study 2, we screened 1149 individuals and enrolled 29 (2.52%; screening details for study 2 are described fully in the study by Leach et al [41]). A total of 86 participants across both studies (study 1: n=57, 66%; study 2: n=29, 34%) completed remote monitoring of physical behavior and were included in the combined data set for analysis. Characteristics of participants included in the combined data set are summarized in Table 1. A comparison of participants in the 2 study samples in terms of demographics, cancer diagnosis, and treatment information is provided in Table S1 in Multimedia Appendix 1. Across studies 1 and 2, the most common cancer types at diagnosis were breast (n=21, 24%), colon (n=20, 23%), and colorectal cancers (n=13, 15%). Detailed information on the distribution of cancer types across the 2 studies is presented in Table S2 in Multimedia Appendix 1.

All participants who underwent remote monitoring had valid activPAL data for least 4 days during the remote monitoring period. Participants had an average of 7.2 (SD 1.4; range 4-13) days of valid data and an average of 35.6 (SD 46.1; range 0-177.8) minutes of nonwear time per day. One participant in study 1 did not complete the FACT-G physical well-being subscale. Due to some in-person assessments being suspended during the COVID-19 pandemic, submaximal VO_2_ values were only available for 37% (21/57) of the participants in study 1. Submaximal VO_2_ values were available for all but 1 participant (28/29, 97%) in study 2 (due to an equipment malfunction). This yielded a total of 49 participants across both studies with available values for submaximal VO_2_.

A summary of the measures of self-reported well-being and physical function, aerobic fitness, and real-world physical behavior is presented in Table 2. Although no ceiling effects were observed for FACT-G total well-being scores, moderate ceiling effects were observed for the FACT-G physical well-being subscale, the FACT-G physical well-being 5-item subset, and linked PROMIS-PF scores, with 19% (16/85), 22% (19/85), and 22% (19/85) of the participants having the maximum score, respectively. Three of the self-reported measures had skewness <–1; distributions of all measures are visualized in Figures S1 and S2 in Multimedia Appendix 1.

As expected, FACT-G total well-being, FACT-G physical well-being, FACT-G physical well-being 5-item subset, and linked PROMIS-PF T scores were significantly correlated (Figure S3 in Multimedia Appendix 1). Similarly, the various measures of real-world physical behavior exhibited mostly expected intercorrelations (Figure S4 in Multimedia Appendix 1).

**Table 1:** Participant characteristics (n=86).

Characteristics			Values
Age (y), mean (SD; range)			55.4 (12.9); 21-85
**Sex, n (%)**			
	Female	61 (71)	
	Male	25 (29)	
BMI, mean (SD; range)			27.4 (5.2; 18-43)
**Education level, n (%)**			
	12th grade or less	0 (0)	
	High school graduate or GED	3 (4)	
	Some college, AA degree, or technical school	21 (24)	
	College graduate (Bachelor’s)	29 (34)	
	Graduate degree (masters or doctorate)	32 (37)	
	Prefer not to answer	1 (1)	
Time since diagnosis (mo), mean (SD; range)			32 (25.5; 2-211)
Time since last treatment (mo), mean (SD; range)			21.2 (17.0; 0-60)
**Cancer stage at diagnosis, n (%)**			
	0^a^	4 (5)	
	I	15 (17)	
	II	22 (26)	
	III	29 (34)	
	IV	9 (11)	
	Unsure	7 (8)	
**Cancer treatment**			
	Had any treatment	86 (100)	
	Had chemotherapy	65 (76)	
	Had radiation	42 (49)	
	Had surgery	76 (88)	
	Had other	12 (14)	
**Number of treatment types, n (%)**			
	1	13 (15)	
	2	41 (47.7)	
	3	28 (32.6)	
	4	4 (4.7)	

^a^Stage 0 indicates evidence of abnormal cells in situ.

**Table 2:** Summary of measures of self-reported well-being and physical function, aerobic fitness, and real-world physical behavior.

Measures		Values, mean (SD; range)
**Self-reported well-being and physical function**		
	FACT-G^a^ total well-being (0-108)	87.9 (13.3; 41-107)
	FACT-G physical well-being subscale (0-28)	24.4 (3.7; 12-28)
	FACT-G physical well-being subscale 5-item subset (0-20)	17.4 (2.6; 9-20)
	Linked PROMIS^b^-Physical Function *T*-score (19-61)	51.1 (7.1; 35-61)
**Aerobic fitness**		
	Predicted submaximal VO_2_^c^ (mL/kg/min)	29.1 (9.9; 10.0-50.0)
**Real-world physical behavior**		
	Daily sedentary time (min)	582.1 (102.8; 295.1-819.4)
	Daily step count	6916.3 (2704.5; 1413-17,501)
	Daily time in light activity (min)	305.1 (97.2; 103.8-551.3)
	Daily time in moderate to vigorous activity (min)	4.0 (6.6; 0.0-58.2)
	Daily time in stepping bouts ≥1 min (min)	25.3 (19.7; 0.2-107.7)
	Weighted median cadence in stepping bouts ≥1 min (steps/min)	98.7 (12.1; 56.5-126.2)
	Peak 30-second cadence (steps/min)	67.2 (8.6; 42.0-86.0)

^a^FACT-G: Functional Assessment of Cancer Therapy-General.

^b^PROMIS: Patient-Reported Outcomes Measurement Information System.

^c^VO_2_: submaximal oxygen uptake.

### Most Measures of Real-World Physical Behavior Were Not Associated With Self-Reported Well-Being or Physical Function

**Figure 1:**
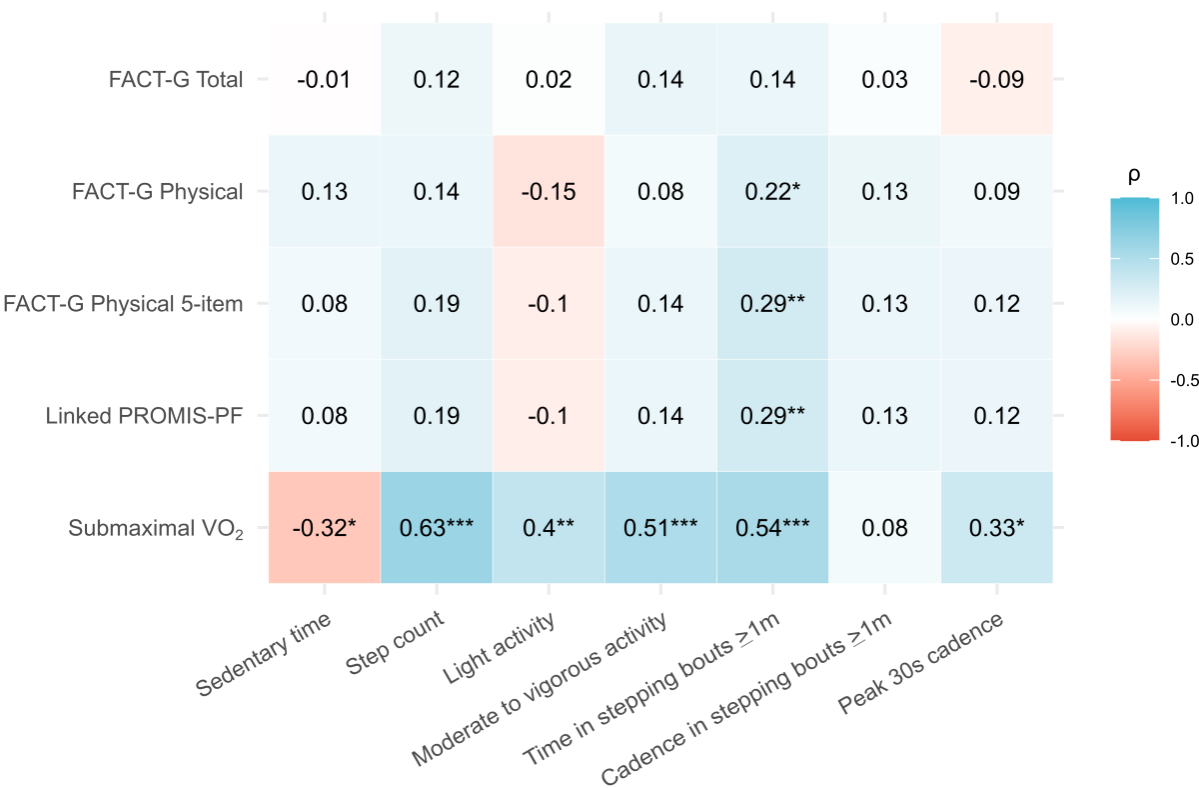
Correlation matrix depicting pairwise Spearman correlations with measures of real-world physical behavior. FACT-G: Functional Assessment of Cancer Therapy-General; PROMIS-PF: Patient-Reported Outcomes Measurement Information System-Physical Function. *P<.05, **P<.01, ***P<.001.

Spearman correlations with real-world physical behavior are depicted in Figure 1. The various measures of real-world physical behavior were not significantly correlated with FACT-G total well-being (*P*s≥.189; section 5 in Multimedia Appendix 1). Average daily time in stepping bouts ≥1 minute was significantly correlated with FACT-G physical well-being (ρ=0.22; *P*=.046), FACT-G physical well-being 5-item subset (ρ=0.29; *P*=.007), and linked PROMIS-PF T scores (ρ=0.29; *P*=.007), but no other measures of physical behavior were associated with FACT-G physical well-being, FACT-G physical well-being 5-item subset, or linked PROMIS-PF T scores (*P*s≥.08; section 5 in Multimedia Appendix 1). When accounting for the effects of demographics and cancer characteristics on these associations using a partial Spearman correlation framework, the pattern of significance was largely unchanged, except that the correlation between time in stepping bouts ≥1 minute and FACT-G physical well-being was no longer significant (Figure S5 in Multimedia Appendix 1).

**Figure 2:**
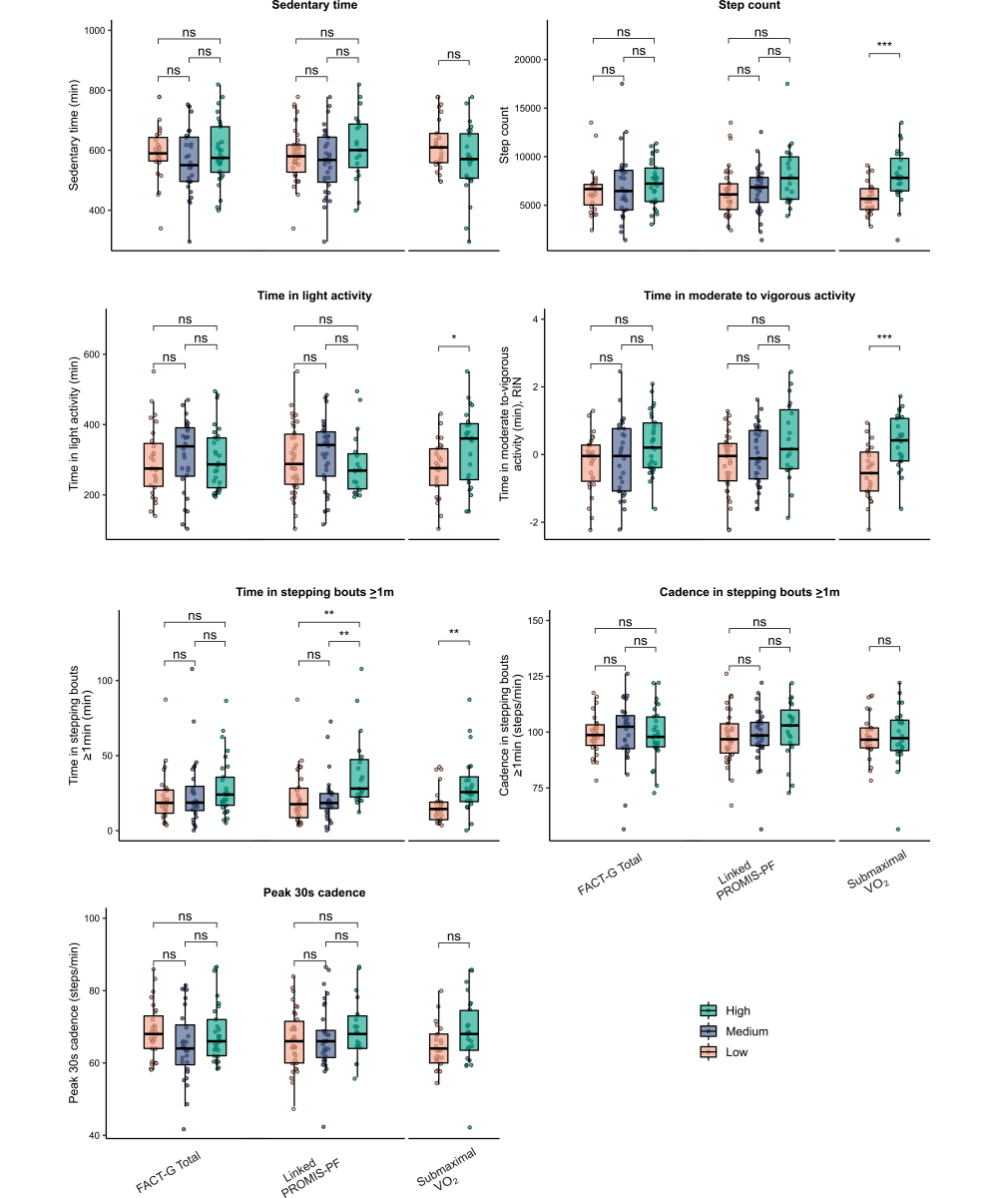
Box plots depicting the measures of real-world physical behavior according to tertile splits of self-reported well-being and physical function and a median split of aerobic fitness. FACT-G: Functional Assessment of Cancer Therapy-General; PROMIS-PF: Patient-Reported Outcomes Measurement Information System-Physical Function; submaximal VO_2_: submaximal oxygen uptake. Significance labels refer to the results of Welch *t* tests and Mann-Whitney *U* tests. For ease of visualization, time in moderate to vigorous activity was transformed with a reverse inverse normal (RIN) transformation. *P<.05, **P<.01, ***P<.001.

Individuals with high, medium, and low FACT-G total well-being scores did not differ significantly in terms of any of the measures of real-world physical behavior (Figure 2). Similarly, individuals with high, medium, and low levels of FACT-G physical well-being scores, FACT-G physical well-being 5-item subset scores, and linked PROMIS-PF T scores did not differ significantly in terms of sedentary time, step counts, time in moderate to vigorous activity, weighted median cadence, or peak 30-second cadence (Figure 2; Figure S6 in Multimedia Appendix 1). However, we did find that participants with high FACT-G physical well-being 5-item subset and linked PROMIS-PF T scores spent more time in stepping bouts ≥1 minute than those with medium (*W*=121; *P*=.001) and low scores (*W*=155; *P*=.004).

### Real-World Physical Behavior Was Associated With Aerobic Fitness

All but one of the accelerometry-derived measures of real-world physical behavior were significantly correlated with submaximal VO_2_ (*P*s≤.03; Figure 1; section 5 in Multimedia Appendix 1). Weighted median cadence in stepping bouts ≥1 minute was the only measure not associated with submaximal VO_2_ (ρ=0.08; *P*=.61). After accounting for the effects of demographics and cancer characteristics in a partial Spearman correlation framework (Figure S5 in Multimedia Appendix 1), average daily step count was significantly correlated with submaximal VO_2_ (ρ=0.46; *P*=.002), as was time in moderate to vigorous activity (ρ=0.33; *P*=.03).

A median split of submaximal VO_2_ (Figure 2) indicated that compared with participants with low submaximal VO_2_, participants with high submaximal VO_2_ had significantly higher step counts (*W*=130; *P*<.001) and spent significantly more time in light intensity activity (t_42.6_=2.23; *P*=.03), moderate intensity activity (*W*=128; *P*<.001), and stepping bouts ≥1 minute in duration (*W*=144; *P*=.002). Individuals with high and low submaximal VO_2_ did not differ significantly in terms of sedentary time (*W*=367; *P=*.19), weighted median cadence (t_42.7_=-0.07; *P=*.95), or peak 30-second cadence (*W*=212; *P=*.08).

### Aerobic Fitness Was Not Associated With Self-Reported Well-Being or Physical Function

Spearman correlation analyses indicated that submaximal VO_2_ was not significantly correlated with any of the self-reported measures (*P*s≥.21; section 7 in Multimedia Appendix 1). The pattern of significance was unchanged when using a partial correlation approach to account for the effects of demographic and cancer characteristics on these associations (*P*s≥.27; section 7 in Multimedia Appendix 1).

### Associations With Real-World Physical Behavior Were Stronger for Aerobic Fitness Than for Self-Reported Well-Being or Physical Function

**Figure 3:**
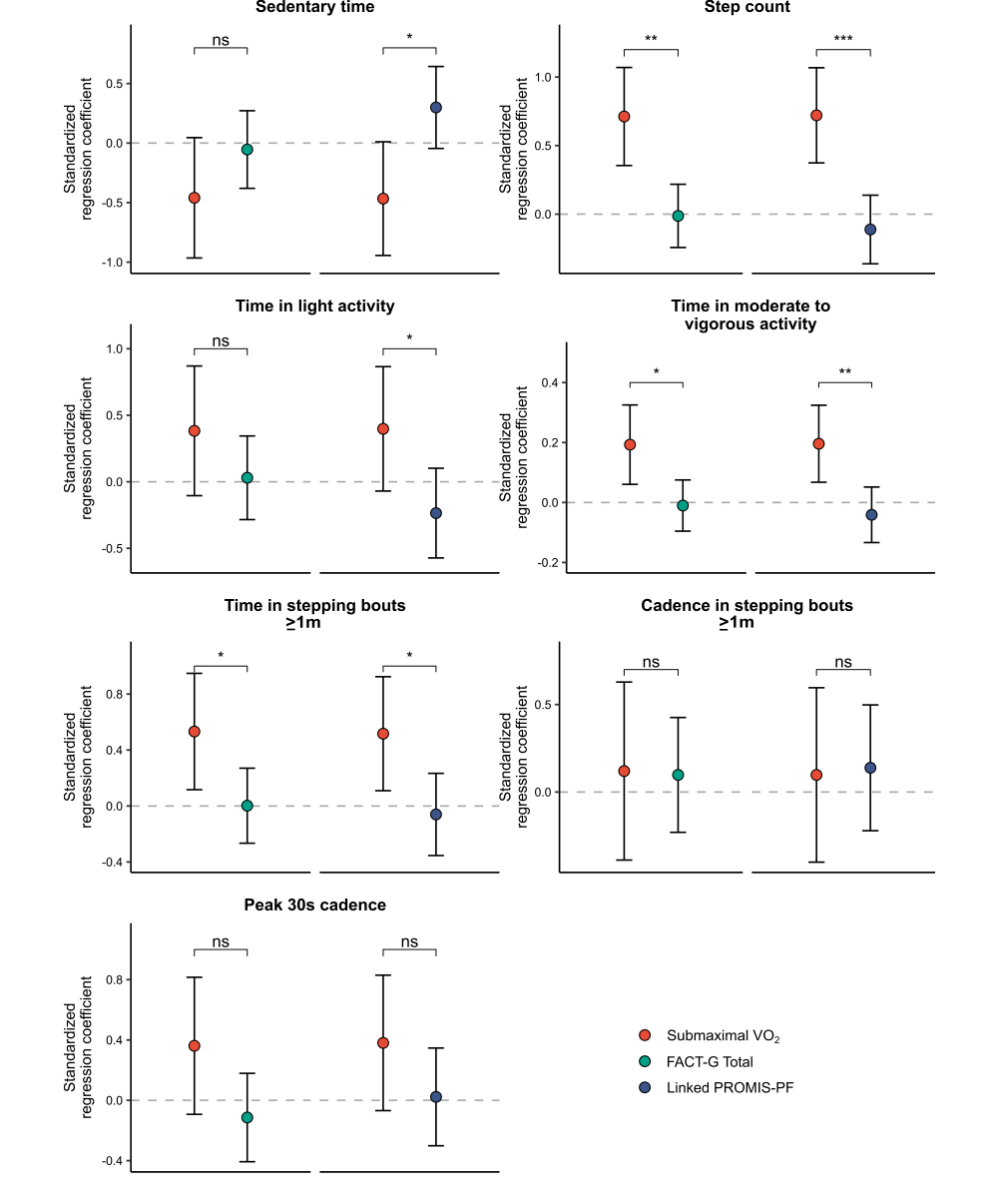
Comparison of associations of real-world physical behavior with (1) self-reported well-being and physical function and (2) aerobic fitness. FACT-G: Functional Assessment of Cancer Therapy-General; PROMIS-PF: Patient-Reported Outcomes Measurement Information System-Physical Function; submaximal VO_2_: submaximal oxygen uptake. Significance labels refer to the results of likelihood ratio (F) tests comparing standardized regression coefficients. *P<.05, **P<.01, ***P<.001.

Having found that the measures of real-world physical behavior were largely uncorrelated with self-reported well-being and physical function but correlated with aerobic fitness, we used likelihood ratio tests to compare these sets of associations (Figure 3; Figure S8 in Multimedia Appendix 1). These analyses indicated that step count was more strongly associated with submaximal VO_2_ than with FACT-G total well-being (*F*_1_=12.29; *P*=.001), FACT-G physical well-being (*F*_1_=18.27; *P*<.001), FACT-G physical well-being 5-item subset (*F*_1_=16.32; *P*<.001), and linked PROMIS-PF T scores (*F*_1_=15.72; *P*<.001). Similarly, time in moderate to vigorous activity was more strongly associated with submaximal VO_2_ than with FACT-G total well-being scores (*F*_1_=7.05; *P*=.01), FACT-G physical well-being (*F*_1_=8.78; *P*=.005), FACT-G physical well-being 5-item subset (*F*_1_=8.13; *P*=.007), and linked PROMIS-PF T scores (*F*_1_=9.30; *P*=.004). Time in stepping bouts ≥1 minute was also more strongly associated with submaximal VO_2_ than with any of the self-reported measures (FACT-G total: *F*_1_=4.87; *P*=.03; FACT-G physical: *F*_1_=8.34; *P*=.006; FACT-G physical 5-item subset: *F*_1_=7.16; *P*=.01; linked PROMIS-PF: *F*_1_=5.48; *P*=.03).

Sedentary time was more negatively associated with submaximal VO_2_ than with FACT-G physical well-being (*F*_1_=7.49; *P*=.009), FACT-G physical well-being 5-item subset (*F*_1_=5.36; *P*=.03), and linked PROMIS-PF T scores (*F*_1_=7.02; *P*=.01), but not with FACT-G total well-being scores (*F*_1_=1.93; *P*=.17). Similarly, time in light activity was more positively associated with submaximal VO_2_ than with FACT-G physical well-being (*F*_1_=4.86; *P*=.03) and linked PROMIS-PF T scores (*F*_1_=5.01; *P*=.03), but not with FACT-G total well-being scores (*F*_1_=1.57; *P*=.22) or physical well-being 5-item subset scores (*F*_1_=4.01; *P*=.05). For weighted median cadence and peak 30-second cadence, associations with submaximal VO_2_ were not significantly different than those with any of the participant-reported measures (*P*s≥.08; section 8 in Multimedia Appendix 1).

A similar pattern of results was observed when examining relationships with measures of activity fragmentation (Multimedia Appendix 1). Specifically, measures indicating a more fragmented activity pattern were correlated with lower submaximal VO_2_ but were largely unrelated to measures of self-reported well-being and physical function (Figure S9 in Multimedia Appendix 1); furthermore, multiple measures of activity fragmentation were significantly more associated with aerobic fitness than with the self-reported measures (Figure S10 in Multimedia Appendix 1).

## Discussion

### Principal Findings

Amid a digital revolution in medicine, the use of digital health technologies as evidence generation tools in oncology clinical trials and routine cancer care is gaining traction [27,67]. Wearable sensors are increasingly being used for assessing the efficacy of anticancer therapies and for posttreatment monitoring [29], but the clinical utility of measures of real-world behavior derived from these devices remains to be fully characterized. In this study, we examined how measures of real-world physical behavior, captured in real-world environments of cancer survivors over a 1-week monitoring period using accelerometry, were related to self-reported and performance outcomes. We found that the volume and patterning of real-world physical behavior were more related to aerobic fitness than to self-reported well-being and physical function.

Previous studies assessing relationships between real-world measures of physical behavior and self-reported well-being and physical function in cancer survivors have reported mixed findings. In a study of prostate cancer survivors, accelerometer-assessed time spent sedentary, time in light activity, and time in moderate to vigorous activity were all associated with global well-being, but only at specific percentiles of well-being [68]. In colon cancer survivors, time spent sedentary was associated with quality of life [69], and among colorectal cancer survivors, time in moderate to vigorous activity was associated with quality of life and physical function [70]. However, one of these studies failed to find a significant association between sedentary time and either quality of life or physical function [70], and in a separate study, neither time in sedentary behavior nor time in moderate to vigorous activity was significantly associated with quality of life in prostate cancer survivors [71]. Our findings are in line with these prior reports of limited relationships with measures of real-world physical behavior and suggest that these measures are more reflective of objective physical capacity than of self-reported well-being and physical function in cancer survivors.

There are several potential explanations for why we did not observe many significant relationships between real-world physical behavior and the self-reported measures. One reason may be that ceiling effects in the self-reported measures limited our ability to detect associations with physical behavior. We observed ceiling effects for FACT-G physical well-being and linked PROMIS-PF T scores, which may be due to selection bias, as well as some participants being far out from diagnosis and treatment at the time of assessment. All participants in studies 1 and 2 had completed treatment, and participants in both the studies had been diagnosed an average of 32 months before data collection. Ceiling effects are a limitation of some participant-reported assessments, including FACT-G, its subscales, and PROMIS-PF short forms [24,54,72,73], with these effects challenging the ability of an assessment to detect changes over time [56]. These effects may be especially relevant when respondents have higher levels of functioning [24,54], which could occur when assessing cancer survivors (1) years out from diagnosis, (2) with cancer types that tend to be detected early, or (3) who experience relatively smaller declines in functioning. As fewer of the real-world physical behavior measures were highly skewed, these measures have the potential to capture aspects of functioning beyond those captured with self-reported measures.

Another reason may be that the real-world measures studied here do not capture the aspects of real-world physical behavior that are most associated with self-reported well-being and physical function. We included a range of measures of real-world physical behavior, with the aim of gaining insights into their differential clinical utility. Step count, time in moderate to vigorous activity, time spent sedentary, time in light activity, and time in stepping bouts ≥1 minute, all demonstrated stronger associations with aerobic fitness than with the self-reported measures, suggesting that these particular measures may offer more insights into individuals’ physical capacity than their well-being and perceived physical function. We found that weighted median cadence and peak 30-second cadence were largely unrelated to aerobic fitness, and their associations with aerobic fitness did not differ from those for the participant-reported measures. It is worth noting that we calculated time and weighted median cadence in stepping bouts ≥1 minute in duration (rather than longer-duration stepping bouts), due to many participants not spending time in longer-duration stepping bouts. As most stepping bouts taken in day-to-day behavior tend to be <1 minute in duration [74], time and cadence in longer-duration stepping bouts may be more informative, but studies of larger samples are needed to examine the clinical utility of these measures. At the same time, with participants spending the most time in short-duration stepping bouts, aspects of gait such as gait speed and variability not explored here may be clinically relevant measures of day-to-day functioning and worth further investigation.

Beyond measures reflecting the absolute volume of physical behavior, we found that measures reflecting a more fragmented pattern of daily activity and sedentary time were negatively correlated with aerobic fitness but were mostly unrelated to self-reported well-being and physical function. Fragmented daily physical activity has been associated with poorer physical function as measured in the clinic, as well as higher fatiguability [63,64]. Additional research is needed to understand whether measures reflecting the fragmentation of real-world physical behavior can provide additional insights into real-world physical function, beyond measures reflecting the absolute volume of physical behavior, in cancer survivors. Taken together, it may be that further research is needed to define and validate measurable concepts and features of real-world physical behavior that are more closely related to perceived physical function.

We note several other important limitations. First, this was a cross-sectional analysis; results may differ if examining relationships with change in real-world physical behavior. Testing whether real-world physical behavior is associated with established measures of physical function and well-being over time will be necessary for establishing clinical validity of these measures. In addition, the sample size was small, with only 49 individuals included in the analyses involving aerobic fitness due to some in-person assessments being suspended during the COVID-19 pandemic. Furthermore, participants were mostly White and female, with high levels of educational attainment, limiting the ecological validity of results. In addition, most participants were diagnosed at cancer stage II or lower, with breast, colon, or rectal cancer, so results may not generalize to survivors of more advanced cancers or of other cancer types. Similarly, this analysis was focused on individuals who had completed treatment, which allowed us to consider questions of clinical utility without the confounding effects of disease and treatment on functioning; however, results may not generalize to individuals undergoing active cancer treatment. Further investigation of real-world physical behavior in larger, more representative samples of individuals during and after cancer treatment is warranted.

Another important limitation is that this was a secondary analysis of previously collected data, and so the studies were not designed to test the questions posed in this investigation. Related to this, it is possible that using other self-report and aerobic fitness measures might have yielded different results. Additional work to probe these relationships with other measures may help inform the clinical utility of wearable-derived digital measures in cancer survivorship.

Beyond these limitations, our findings speak to the potential utility of digital measures of real-world physical behavior to contribute to the assessment of functioning in cancer survivorship. That the digital measures did not exhibit many significant relationships with self-reported well-being and physical function suggests that these sets of measures provide different information. Furthermore, real-world physical behavior was significantly associated with submaximal VO_2_; if further investigation reveals significant overlap in the clinical utility of these measures, wearable sensors could provide a lower-burden means of capturing information on aerobic fitness. Finally, compared with using any single type of measure, combining participant-reported, performance, and objective real-world measures could provide a more holistic picture of functioning in cancer survivorship [75]. Taking a comprehensive approach to assessing functioning could furthermore increase sensitivity to detect clinical change over time, enabling more efficient discovery of novel anticancer therapeutics or efficacious interventions for cancer survivors. This approach furthermore offers the possibility to better predict clinical outcomes, which could enable earlier disease detection and the personalization of both treatment and survivorship care [76,77]. As they can be captured remotely and passively, digital measures of real-world physical behavior can also enable decentralization of clinical trials, lower patient burden for participation, and facilitate the recruitment of underrepresented populations [78].

Although our findings indicate that digital measures of real-world physical behavior may add value for the measurement of functioning in cancer survivorship, further research is needed to evaluate the relative value and unique contributions of real-world physical behavior and self-reported physical function to the well-being of cancer survivors. Our approach and previous studies have been limited to cross-sectional analyses, but further work assessing how measures of real-world physical behavior relate to established clinical outcomes over time will be important for advancing the appropriate use of digital measures in oncology clinical research [79,80]. There are additional challenges with implementing wearable sensors in clinical populations, including acceptability and feasibility of these devices among participants. In addition, there is a growing regulatory emphasis on patient centricity in the development of clinical outcome assessments, such that digital measures derived from wearable sensors should reflect aspects of health that are meaningful to individuals in the target clinical population of interest [81]. Our findings suggest that digital measures may provide additional insights into physical function beyond those obtained with self-reported assessments, but whether these insights reflect aspects of everyday functioning that are meaningful remains to be determined. Gathering the evidence needed to demonstrate that digital measures are validated, meaningful, and feasible to capture will be important for promoting broad acceptance and proper use of digital measures in oncology clinical research [79,81,82].

### Conclusions

Digital health technologies such as wearable sensors are increasingly used in oncology clinical research and offer potential for capturing aspects of real-world functioning in cancer survivors. In this secondary analysis, we investigated the clinical utility of accelerometry-derived measures of real-world physical behavior in a sample of individuals who had completed cancer treatment. We found that several measures of real-world physical behavior were more associated with aerobic fitness, assessed with a submaximal exercise test, than they were with self-reported measures of well-being and physical function. Our findings suggest that in cancer survivors who have completed treatment, measures of real-world physical behavior may be able to complement self-reported measures of well-being and physical function.

## Appendix

Multimedia Appendix 1 Supplementary methods and results.

## Abbreviations

FACT-G: Functional Assessment of Cancer Therapy-General
PROMIS-PF: Patient-Reported Outcomes Measurement Information System-Physical Function

## Data Availability

The data sets generated and analyzed during this study are available from the corresponding author on reasonable request.
