# Supplementary material for: Do Measures of Real-World Physical Behavior Provide Insights Into the Well-Being and Physical Function of Cancer Survivors? Cross-Sectional Analysis
Source: JMIR Cancer. 2024 Jul 15;10:e53180. doi: 10.2196/53180 (PMC11287100; doi:10.2196/53180)
Supplement: Multimedia Appendix 1 [file cancer_v10i1e53180_app1.pdf]

Supplementary Material

Do measures of real-world physical behavior provide insights into the well-being and physical function of cancer survivors?: A cross-sectional analysis

Shelby L. Bachman[1]*, Emma Gomes[2], Suvekshya Aryal[1], David Cella[3], Ieuan Clay[1], Kate Lyden[1], Heather Leach[2]

[1]VivoSense, Inc., Newport Coast, CA, USA
[2]Colorado State University, Fort Collins, CO, USA
[3]Northwestern University Feinberg School of Medicine, Chicago, IL, USA

*Please address correspondence to:
Shelby L. Bachman
shelby.bachman@vivosense.com

Table of Contents

Supplementary Methods 1
Section 1. Calculation and analysis of activity fragmentation 1
Supplementary Results 2
Section 1. Participant characteristics by study 2
Section 2. Distributions of measures 4
Section 3. Correlations between self-reported measures 6
Section 4. Correlations between measures of real-world physical behavior 7
Section 5. Correlations with real-world measures of physical behavior 8
Section 5. Partial correlations with real-world measures of physical behavior 9
Section 6. Split comparisons of measures of real-world physical behavior 10
Section 7. Correlations between aerobic fitness and the self-reported measures 11
Section 8. Comparison of associations of real-world physical behavior with the self-reported measures and aerobic fitness 12
Section 9. Results of activity fragmentation analyses 14
Supplementary References 18

Supplementary Methods

Section 1. Calculation and analysis of activity fragmentation

Calculation of activity fragmentation measures

For analysis of activity fragmentation, each participant's raw acceleration data were first resampled from 20 to 30Hz. Resampled data were then converted to minute-level activity counts using the R package activityCounts (Version 0.2.0; [1]). For each participant, multiple measures reflecting activity fragmentation across the remote monitoring period were calculated from activity counts using the R package ActFrag (Version 0.1.1; [2]). For this analysis, minutes in which more than 50% of samples were classified as non-wear or primary lying were excluded.

Measures of activity fragmentation included a range of non-parametric and parametric measures [3]. Non-parametric measures included: the mean duration of active and sedentary bouts (μ_a and μ_s, respectively), the Gini index for active and for sedentary bouts (g_a and g_s, respectively), and the average hazard reflecting the probability of transitioning from active to sedentary states and from sedentary to active states (h_a and h_s, respectively). As parametric measures, α_a and α_s were calculated, with these being scaling parameters of the power law distribution reflecting the pattern with which total active and total sedentary time were accumulated, respectively.

Analysis of associations with activity fragmentation

We used pairwise Spearman correlations to test associations between the activity fragmentation measures and each of the self-reported measures, as well as aerobic fitness. In addition, we used a series of pairwise partial Spearman correlation analyses to test these associations after accounting for the effects of age, sex, BMI, cancer stage at diagnosis, and time since diagnosis. A similar approach as described in the main text (see Methods section "Comparison of associations with measures of real-world physical behavior") was used to determine whether activity fragmentation was more associated with the self-reported measures or with aerobic fitness.

Supplementary Results

Section 1. Participant characteristics by study

Table S1

Comparison of demographics and cancer characteristics of participants from Studies 1 and 2.

	Study 1			Study 2			P^a
	N (%)	Mean (SD)	Range	N (%)	Mean (SD)	Range	
**Age (years)**		52.7 (12.8)	21 - 75		60.8 (11.4)	41 - 85	.004
**Sex**							.041
Female	45 (78.9%)			16 (55.2%)			
Male	12 (21.1%)			13 (44.8%)			
**Body mass index**		26.5 (4.9)	18 - 43		29.1 (5.6)	19 - 42	.040
**Education level**							.087
12th grade or less	0 (0%)			0 (0%)			
High school graduate or GED	0 (0%)			3 (10.3%)			
Some college, AA degree, or technical school	12 (21.1%)			9 (31%)			
College graduate (Bachelor's)	21 (36.8%)			8 (27.6%)			
Graduate degree (Master's or doctorate)	23 (40.4%)			9 (31%)			
Prefer not to answer	1 (1.8%)			0 (0%)			
**Time since diagnosis (months)**		37.2 (28.1)	2 - 211		22 (15.1)	4 - 58	.002
**Time since last treatment (months)**		24.5 (17.5)	0 - 60		14.6 (14.3)	1 - 51	.007
**Cancer stage at diagnosis**							.001
0[b]	4 (7%)			0 (0%)			
I	15 (26.3%)			0 (0%)			
II	13 (22.8%)			9 (31%)			
III	12 (21.1%)			17 (58.6%)			
IV	7 (12.3%)			2 (6.9%)			
Unsure	6 (10.5%)			1 (3.4%)			
**Cancer treatment**							
Had treatment	57 (100%)			29 (100%)			
Had chemotherapy	38 (66.7%)			27 (93.1%)			
Had radiation	33 (57.9%)			9 (31%)			
Had surgery	50 (87.7%)			26 (89.7%)			
Had other	12 (21.1%)			0 (0%)			
**Number of treatment types**							.087
1	10 (17.5%)			3 (10.3%)			
2	22 (38.6%)			19 (65.5%)			
3	21 (36.8%)			7 (24.1%)			
4	4 (7%)			0 (0%)			

Note. [a]Comparison *P* values were obtained from Welch's *t*-tests (for continuous variables) or Chi-squared tests of independence (for categorical variables) performed to compare the relevant variable in each study sample. [b]Stage 0 indicates evidence of abnormal cells *in situ*.

Table S2

Cancer type at diagnosis for participants in Study 1, Study 2, and the combined dataset.

Cancer type at diagnosis	N (%)		
	Study 1	Study 2	Combined dataset
Brain & nervous system	3 (5.3%)	0 (0%)	3 (3.5%)
Breast	21 (36.8%)	0 (0%)	21 (24.4%)
Colon	0 (0%)	20 (69%)	20 (23.3%)
Colorectal	9 (15.8%)	4 (13.8%)	13 (15.1%)
Head & neck	1 (1.8%)	0 (0%)	1 (1.2%)
Leukemia	1 (1.8%)	0 (0%)	1 (1.2%)
Lung	3 (5.3%)	0 (0%)	3 (3.5%)
Lymphoma	6 (10.5%)	0 (0%)	6 (7%)
Melanoma	1 (1.8%)	0 (0%)	1 (1.2%)
Other	10 (17.5%)	0 (0%)	10 (11.6%)
Pancreatic	1 (1.8%)	0 (0%)	1 (1.2%)
Rectal	0 (0%)	5 (17.2%)	5 (5.8%)
Thyroid	1 (1.8%)	0 (0%)	1 (1.2%)

Section 2. Distributions of measures

Figure S1

Distributions of measures of self-reported well-being and physical function and aerobic fitness.

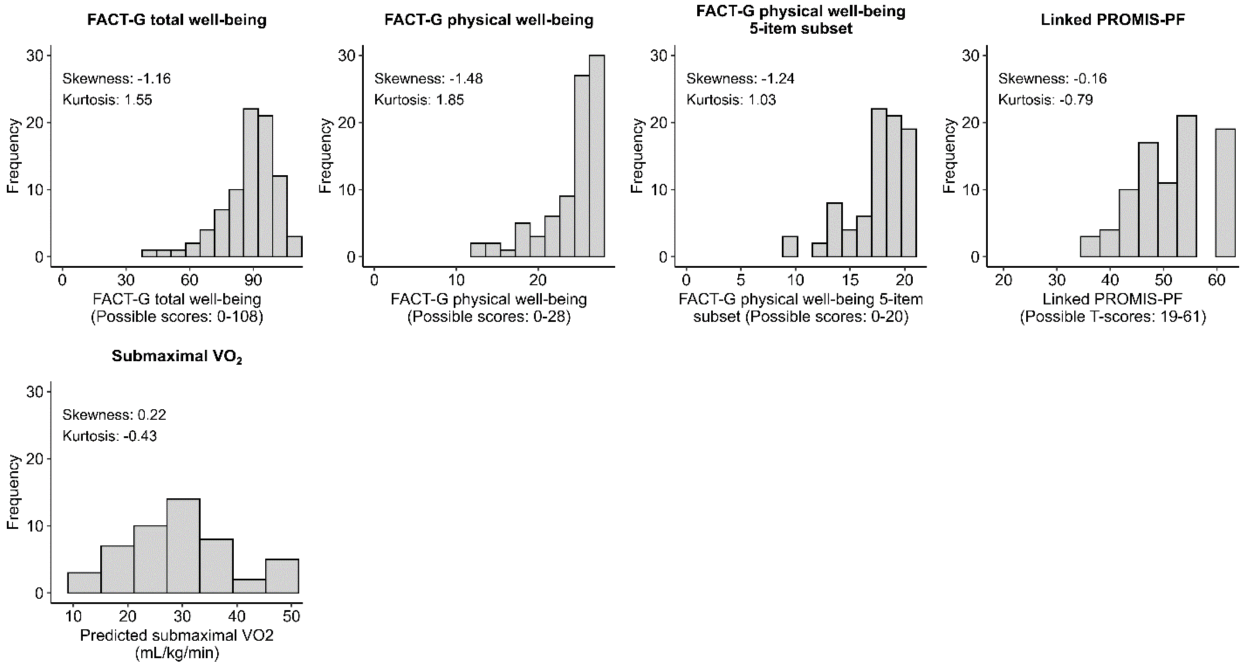

Note. FACT-G = Functional Assessment of Cancer Therapy – General; PROMIS-PF = Linked Patient-Reported Outcomes Measurement Information System – Physical Function.

Figure S2

Distributions of measures of real-world physical behavior.

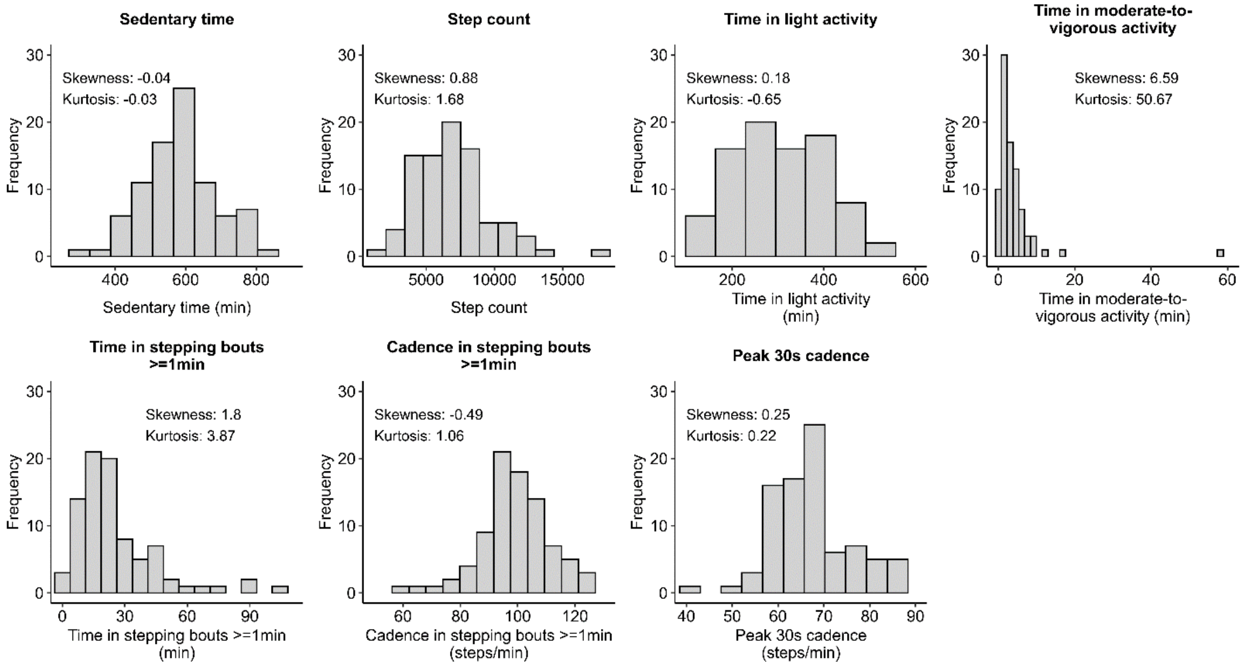

Section 3. Correlations between self-reported measures

Figure S3

Correlation matrix depicting Spearman correlations between self-reported measures.

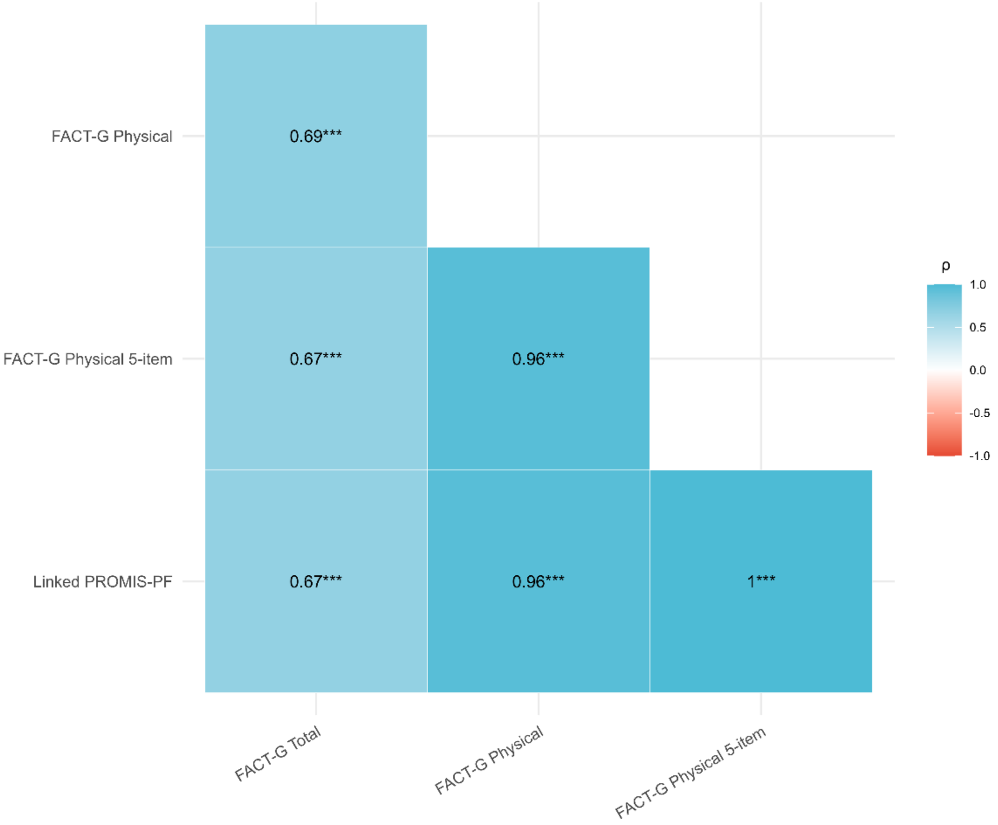

Note. FACT-G = Functional Assessment of Cancer Therapy – General; ns = not significant; PROMIS-PF = Linked Patient-Reported Outcomes Measurement Information System – Physical Function. *$P < .05$, **$P < .01$, ***$P < .001$

Section 4. Correlations between measures of real-world physical behavior

Figure S4

Correlation matrix depicting Spearman correlations between measures of real-world physical behavior.

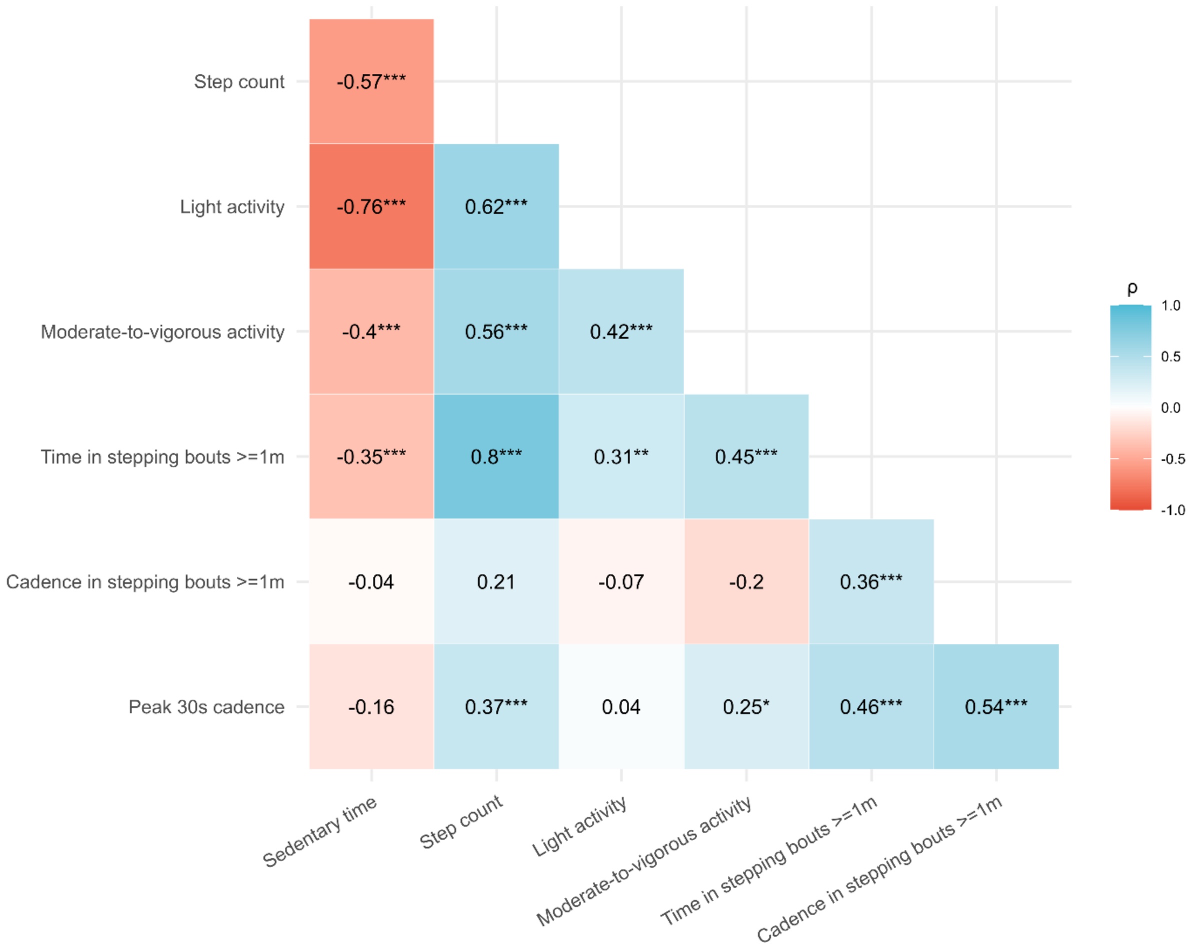

Note. **P* < .05, ***P* < .01, ****P* < .001

Figure S4 depicts correlations between the various measures of real-world physical behavior. Average daily step count, time in sedentary behavior, time in light activity, time in moderate-to-vigorous activity, and time in stepping bouts over 1 minute all exhibited significant intercorrelations (*P*s ≤ .003). Weighted median cadence in stepping bouts over 1 minute exhibited significant correlations with 2 of the measures of real-world behavior (*P*s < .001) but was not significantly correlated with sedentary time (ρ = -0.036, *P* = .741), time in light activity (ρ = -0.066, *P* = .548), time in moderate-to-vigorous activity (ρ = -0.204, *P* = .060), or step count (ρ = 0.210, *P* = .053). Peak 30s cadence was significantly with 4 measures of real-world behavior (*P*s ≤ .018) but was not significantly correlated with sedentary time (ρ = -0.161, *P* = .139) or time in light activity (ρ = 0.043, *P* = .692).

Section 5. Correlations with real-world measures of physical behavior

The various measures of real-world physical behavior were not significantly correlated with FACT-G total well-being (sedentary time: ρ = -0.011, *P* = .920; step count: ρ = 0.120, *P* = .277; time in light activity: ρ = 0.017, *P* = .876; time in moderate-to-vigorous activity: ρ = 0.145, *P* = .189; time in stepping bouts ≥1m: ρ = 0.137, *P* = .214; cadence in stepping bouts ≥1m: ρ = 0.033, *P* = .764; peak 30s cadence: ρ = -0.086, *P* = .437).

Average daily time in stepping bouts ≥1 minute was significantly correlated with FACT-G physical well-being (ρ = 0.217; *P* = .046), FACT-G physical well-being 5-item subset (ρ = 0.288; *P* = .007), and linked PROMIS-PF T scores (ρ = 0.288; *P* = .007). No other measures of physical behavior were associated with FACT-G physical well-being (sedentary time: ρ = 0.128, *P* = .242; step count: ρ = 0.135, *P* = .218; time in light activity: ρ = -0.154, *P* = .159; time in moderate-to-vigorous activity: ρ = 0.078, *P* = .480; cadence in stepping bouts ≥1m: ρ = 0.132, *P* = .228; peak 30s cadence: ρ = 0.086, *P* = .435) or FACT-G physical well-being 5-item subset scores (sedentary time: ρ = 0.082, *P* = .458; step count: ρ = 0.189, *P* = .084; time in light activity: ρ = -0.099, *P* = .366; time in moderate-to-vigorous activity: ρ = 0.139, *P* = .205; cadence in stepping bouts ≥1m: ρ = 0.127, *P* = .246; peak 30s cadence: ρ = 0.124, *P* = .257). Likewise, no other measures of physical behavior were associated with linked PROMIS-PF T scores (sedentary time: ρ = .082, *P* = .458; step count: ρ = 0.189, *P* = .084; time in light activity: ρ = -0.099, *P* = .366; time in moderate-to-vigorous activity: ρ = 0.139, *P* = .205; cadence in stepping bouts ≥1m: ρ = 0.127, *P* = .246; peak 30s cadence: ρ = 0.124, *P* = .257).

All but one of the accelerometry-derived measures of real-world physical behavior were significantly correlated with submaximal VO_2 (sedentary time: ρ = -0.318, *P* = .026; step count: ρ = 0.627, *P* < .001; time in light activity: ρ = 0.395, *P* = .005; time in moderate-to-vigorous activity: ρ = 0.508, *P* < .001; cadence in stepping bouts ≥1m: ρ = 0.075, *P* = .608; peak 30s cadence: ρ = 0.327, *P* = .022)

Section 5. Partial correlations with real-world measures of physical behavior

Figure S5

Correlation matrix depicting partial Spearman correlations between measures of real-world physical behavior, the self-reported measures, and aerobic fitness, after accounting for effects of age, sex, body mass index, time since diagnosis, and cancer stage at diagnosis.

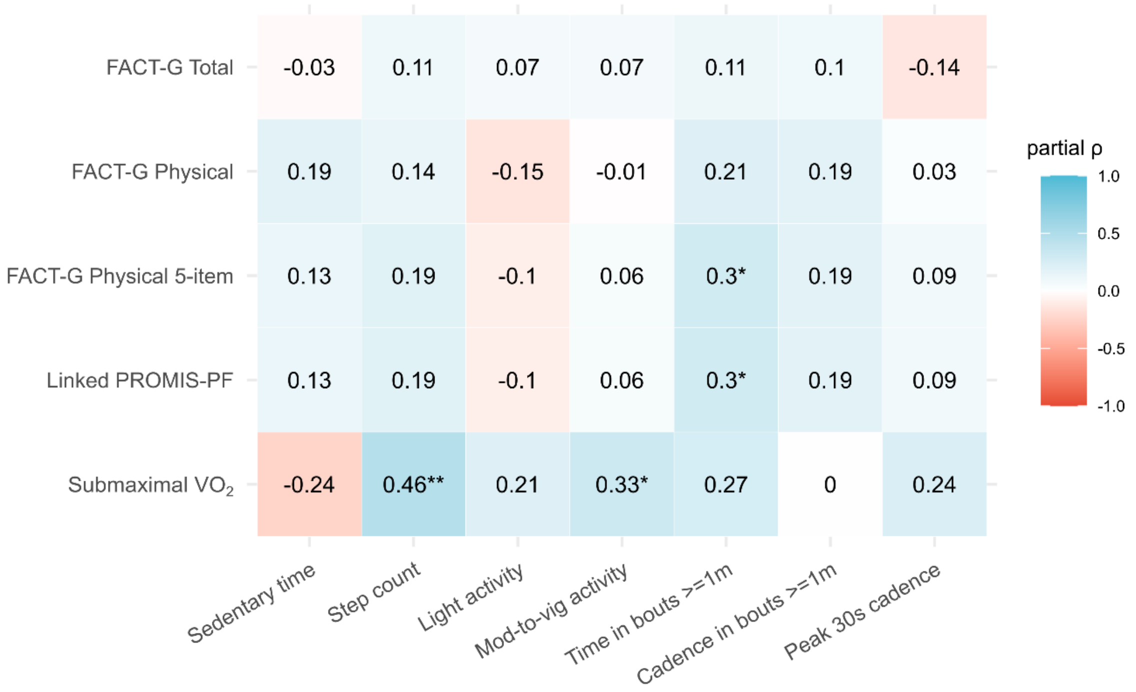

Note. FACT-G = Functional Assessment of Cancer Therapy – General; ns = not significant; PROMIS-PF = Linked Patient-Reported Outcomes Measurement Information System – Physical Function. **P* < .05, ***P* < .01, ****P* < .001

Using a partial correlation approach (Figure S5), the correlation between time in stepping bouts over 1 minute and linked PROMIS-PF T-scores remained significant in the partial correlation framework (ρ = 0.298, *P* = .011), as did the correlation between time in stepping bouts over 1 minute and FACT-G physical well-being 5-item subset scores (ρ = 0.298, *P* = .011). However, the correlation between time in stepping bouts over 1 minute and FACT-G physical well-being was no longer significant (ρ = 0.213, *P* = .070). Using the partial correlation approach, no other measures of physical behavior were significantly correlated with FACT-G total or physical well-being, FACT-G physical well-being 5-item subset, or linked PROMIS-PF T-scores (*P*s ≥ .101; Figure S5).

Section 6. Split comparisons of measures of real-world physical behavior

Figure S6

Boxplots depicting measures of real-world physical behavior according to tertile splits of FACT-G physical well-being and the FACT-G physical well-being 5-item subset.

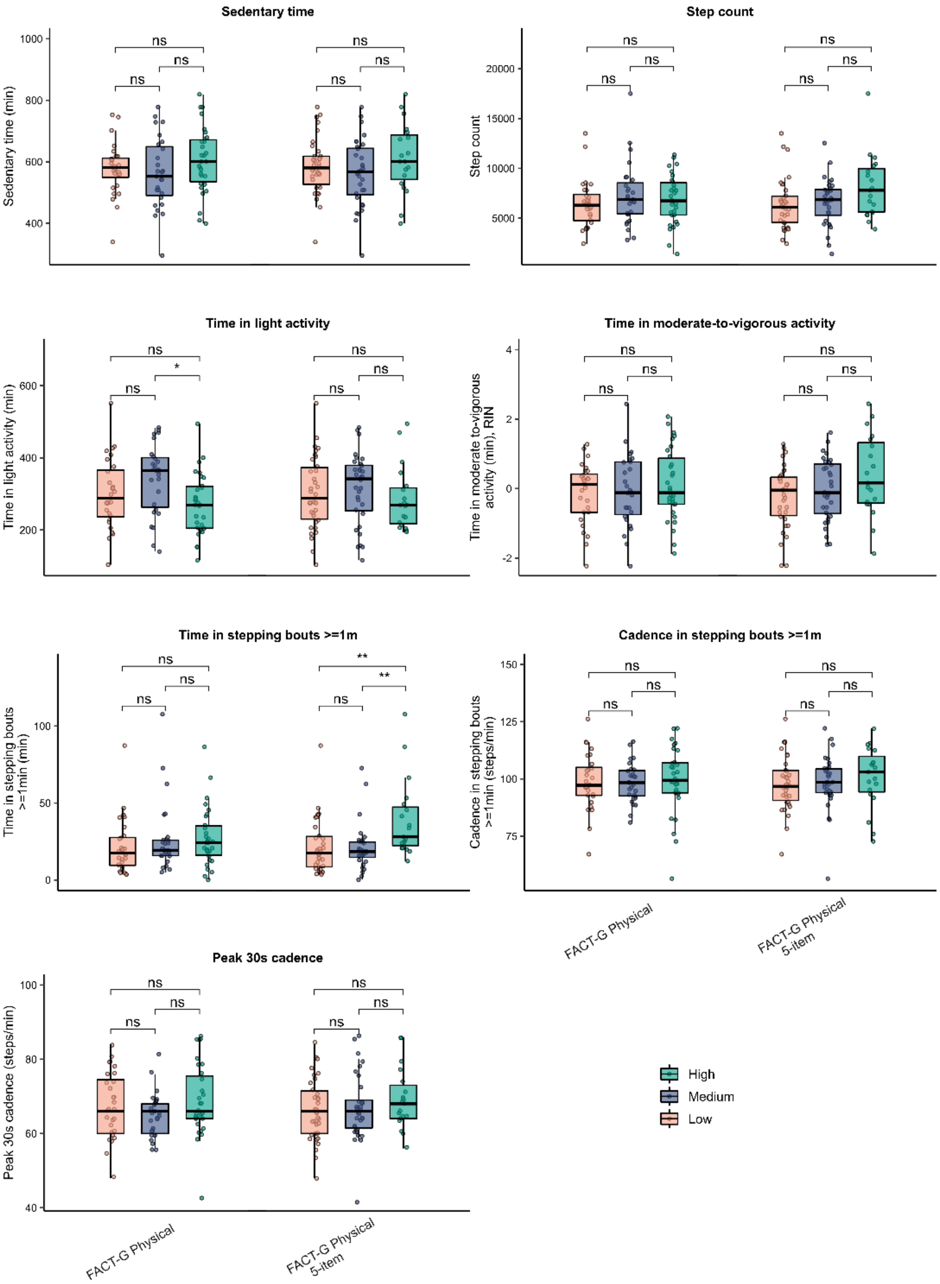

Note. Significance labels refer to the results of Welch's t-tests and Mann-Whitney U-tests. For ease of visualization, time in moderate-to-vigorous activity was transformed with a reverse inverse normal (RIN) transformation. FACT-G = Functional Assessment of Cancer Therapy – General; ns = not significant; PROMIS-PF = Linked Patient-Reported Outcomes Measurement Information System – Physical Function. *P < .05, **P < .01, ***P < .001

Section 7. Correlations between aerobic fitness and the self-reported measures

Spearman correlation analyses indicated that submaximal VO_2 was not significantly correlated with any of the self-reported measures (Figure S7A; FACT-G total well-being: $\rho = .098$, $P = .513$; FACT-G physical well-being: $\rho = 0.157$, $P = .286$; FACT-G physical well-being 5-item subset: $\rho = 0.184$, $P = .211$; linked PROMIS-PF: $\rho = 0.184$, $P = .211$). The pattern of significance was unchanged when using a partial correlation approach to account for the effects of demographic and cancer characteristics on these associations (Figure S7B; FACT-G total well-being: $\rho = 0.034$, $P = .836$; FACT-G physical well-being: $\rho = 0.136$, $P = .396$; FACT-G physical well-being 5-item subset: $\rho = 0.177$, $P = .268$; linked PROMIS-PF: $\rho = 0.177$, $P = .268$).

Figure S7

Correlation matrices depicting pairwise Spearman correlations between aerobic fitness and the self-reported measures of well-being and physical function (A), and partial Spearman correlations after accounting for effects of age, sex, body mass index, time since diagnosis, and cancer stage at diagnosis (B).

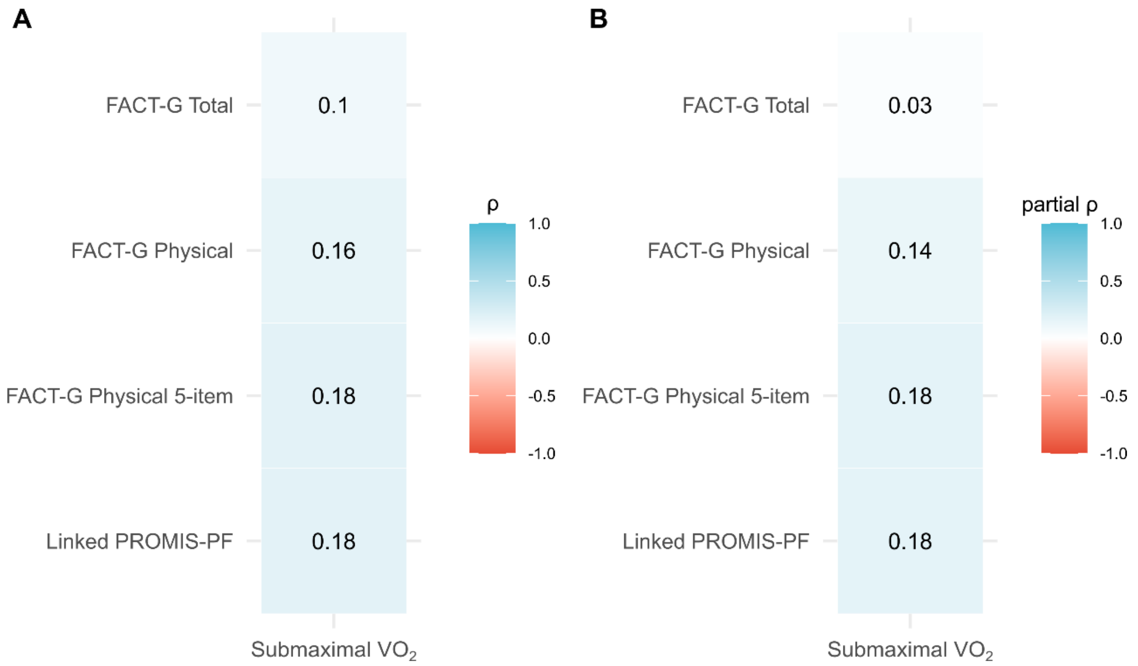

Note. FACT-G = Functional Assessment of Cancer Therapy – General; ns = not significant; PROMIS-PF = Linked Patient-Reported Outcomes Measurement Information System – Physical Function. $*P < .05$, $**P < .01$, $***P < .001$

Section 8. Comparison of associations of real-world physical behavior with the self-reported measures and aerobic fitness

Figure S8

Comparison of associations of real-world physical behavior with (1) self-reported well-being and physical function and (2) aerobic fitness.

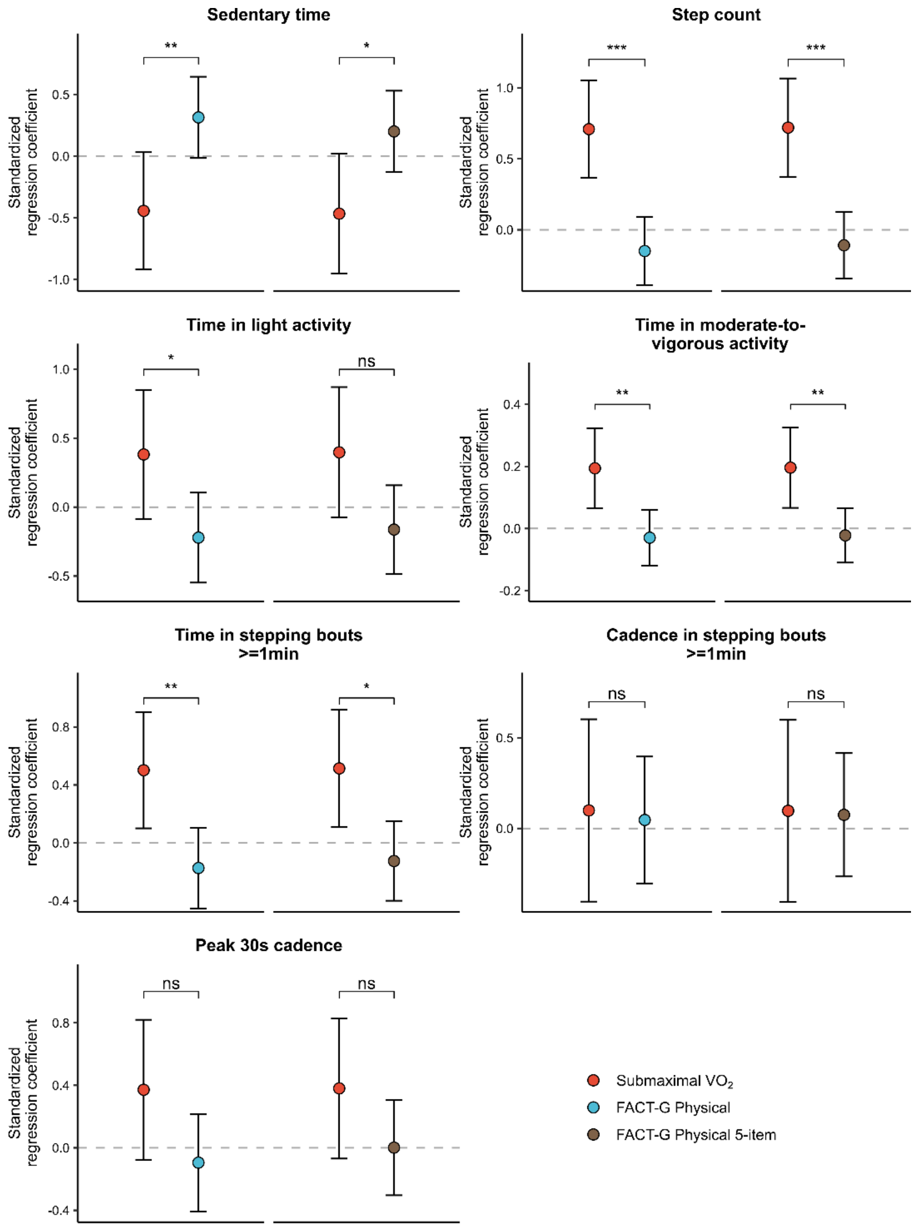

Note. Significance labels refer to the results of likelihood ratio (*F*) tests comparing standardized regression coefficients. FACT-G = Functional Assessment of Cancer Therapy – General; ns = not significant; PROMIS-PF = Linked Patient-Reported Outcomes Measurement Information System – Physical Function. $*P < .05$, $**P < .01$, $***P < .001$

For weighted median cadence in stepping bouts ≥1m, associations with submaximal VO_2 were not significantly different than those with any of the participant-reported measures (FACT-G total well-being: $F_1 = 0.01$, $P = .941$; FACT-G physical well-being: $F_1 = 0.03$, $P = .859$; FACT-G physical well-being 5-item subset: $F_1 = 0.01$, $P = .941$; linked PROMIS-PF: $F_1 = 0.02$, $P = .893$). For peak 30-second cadence, associations with submaximal VO_2 were also not significantly different than those with any of the participant-reported measures (FACT-G total well-being: $F_1 = 3.28$, $P = .078$; FACT-G physical well-being: $F_1 = 3.19$, $P = .082$; FACT-G physical well-being 5-item subset: $F_1 = 2.04$, $P = .162$; linked PROMIS-PF: $F_1 = 1.74$, $P = .196$).

Section 9. Results of activity fragmentation analyses

Activity fragmentation was associated with aerobic fitness, but not with self-reported well-being or physical function

Figure S9

Correlation matrices depicting (A) Spearman correlations and (B) partial Spearman correlations between measures of activity fragmentation, self-reported well-being and physical function, and aerobic fitness.

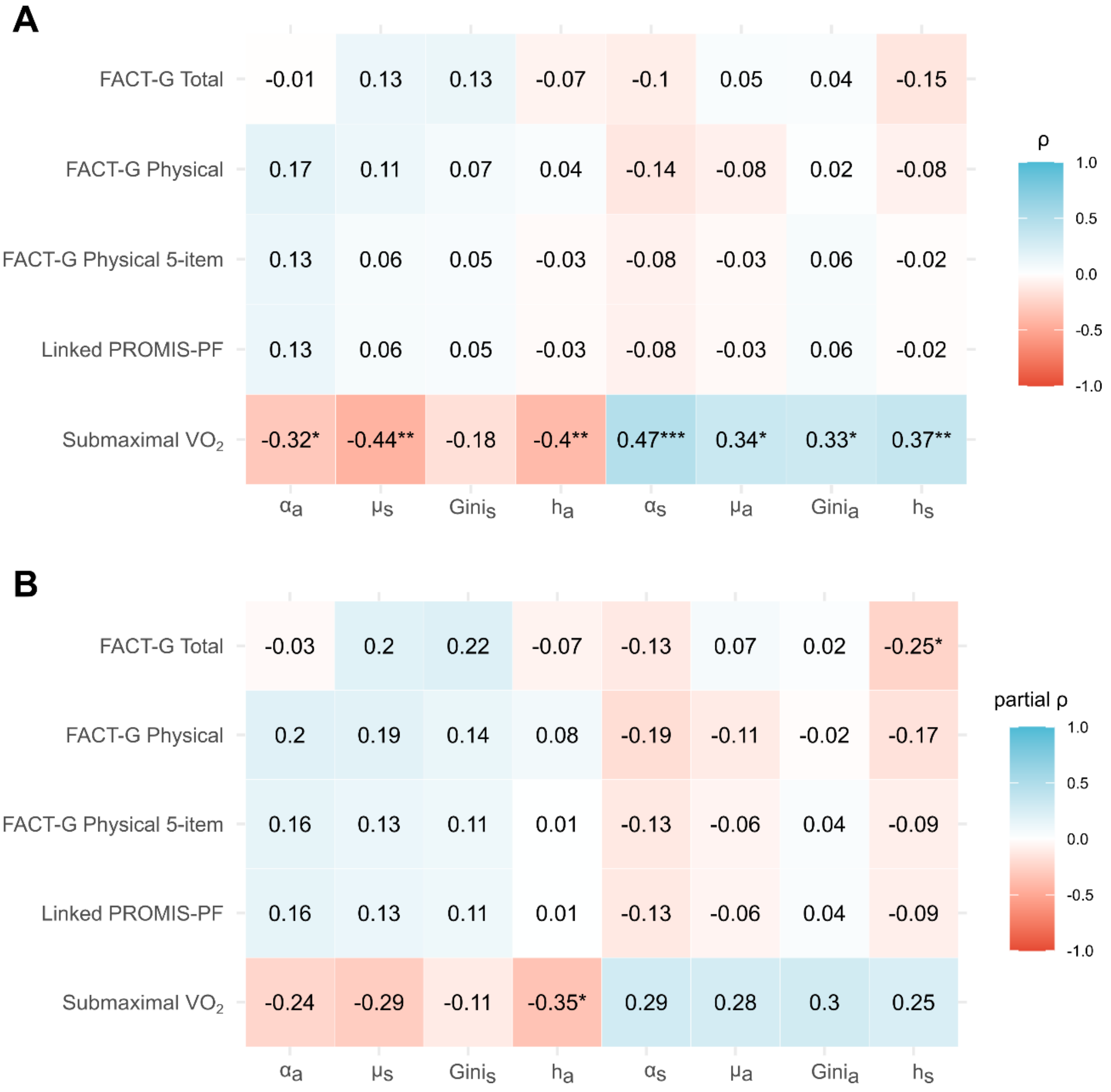

Note. α_a = scaling parameter of the power law for active bouts; α_s = scaling parameter of the power law for sedentary bouts; μ_a = mean duration of active bouts; μ_s = mean duration of sedentary bouts; g_a = Gini index for active bouts; g_s = Gini index for sedentary bouts; h_a = average hazard reflecting the probability of transitioning from active to sedentary states; h_s = average hazard reflecting the probability of transitioning

from sedentary to active states; FACT-G = Functional Assessment of Cancer Therapy – General; PROMIS-PF = Linked Patient-Reported Outcomes Measurement Information System – Physical Function. *$P < .05$, **$P < .01$, ***$P < .001$

None of the calculated activity fragmentation measures were significantly correlated with FACT-G total well-being, FACT-G physical well-being, FACT-G physical well-being 5-item subset, or linked or linked PROMIS-PF T-scores (Figure S9A; *P*s $\geq .110$). Using a partial Spearman correlation framework to account for the effects of age, sex, BMI, time since diagnosis, and cancer stage at diagnosis on these relationships, the pattern of significance was similar (Figure S9B), with the exception that FACT-G total well-being was negatively correlated with the probability of transitioning from a sedentary to active state ($\rho = -0.250$, $P = .034$). In the partial correlation framework, there were no other significant correlations between the self-reported measures and measures of activity fragmentation (Figure S9B; *P*s $\geq .610$).

In contrast, 7 of the 8 investigated measures of activity fragmentation were significantly correlated with submaximal VO_2 (Figure S9A). Specifically, higher submaximal VO_2 was negatively correlated with α_a ($\rho = -0.318$, $P = .026$), mean duration of sedentary bouts (u_s; $\rho = -0.441$, $P = .002$), and the probability of transitioning from active to sedentary bouts (h_a; $\rho = -0.401$, $P = .004$). In addition, submaximal VO_2 was positively correlated with α_r ($\rho = 0.473$, $p < .001$), mean duration of active bouts μ_a ($\rho = 0.343$, $P = .016$), Gini index for active bouts (g_a; $\rho = 0.329$, $P = .021$), and the probability of transitioning from sedentary to active states h_s ($\rho = 0.372$, $P = .009$). Using a partial correlation approach (Figure S9B), only the negative correlation between submaximal VO_2 and h_a remained significant ($\rho = -0.346$, $P = .024$).

Activity fragmentation was more associated with aerobic fitness than with self-reported well-being and physical function

Figure S10

Comparing associations of activity fragmentation with (1) self-reported well-being and physical function and (2) aerobic fitness.

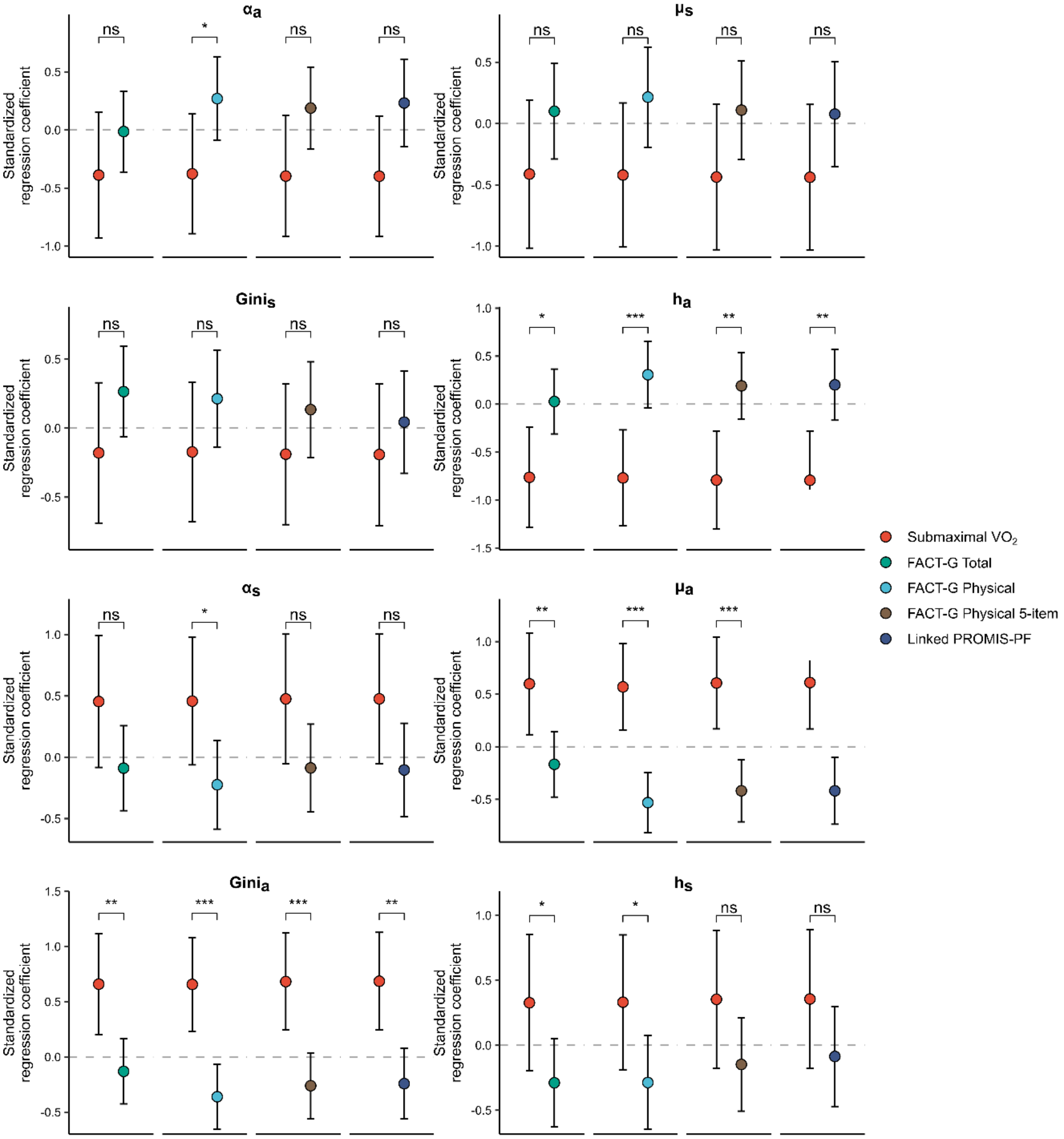

Note. Significance labels refer to the results of likelihood ratio (*F*) tests comparing standardized regression coefficients. α_a = scaling parameter of the power law for active bouts; α_s = scaling parameter of the power law for sedentary bouts; μ_a = mean duration of active bouts; μ_s = mean duration of sedentary bouts; g_a = Gini index for active bouts; g_s = Gini index for sedentary bouts; h_a = average hazard reflecting the

probability of transitioning from active to sedentary states; h_s = average hazard reflecting the probability of transitioning from sedentary to active states; FACT-G = Functional Assessment of Cancer Therapy – General; ns = not significant; PROMIS-PF = Linked Patient-Reported Outcomes Measurement Information System – Physical Function. $^*P < .05$, $^{**}P < .01$, $^{***}P < .001$

Results of likelihood ratio tests used to determine whether the measures of activity fragmentation were more associated with submaximal VO_2 or with measures of self-reported well-being and physical function are shown in Figure S10. These analyses indicated that the mean duration of active bouts (μ_a) was significantly more associated with submaximal VO_2 than with FACT-G total well-being ($F_1 = 7.51$, $P = .009$), FACT-G physical well-being ($F_1 = 21.05$, $P < .001$), FACT-G physical well-being 5-item subset ($F_1 = 15.81$, $P < .001$), and linked PROMIS-PF scores ($F_1 = 14.87$, $P < .001$). In addition, the probability of transitioning from active to sedentary states (h_a) and the Gini index for active bouts (g_a) were both more related to submaximal VO_2 than to any of the self-reported measures (FACT-G total well-being: Ps $\leq .013$; FACT-G physical well-being: Ps < 001; FACT-G physical well-being 5-item subset: Ps $\leq .002$; linked PROMIS-PF scores: Ps < .001).

The scaling parameters of the power law distribution for active and sedentary bouts (α_a and α_s) were both more associated with submaximal VO_2 than with FACT-G physical well-being (α_a: $F_1 = 4.64$, $P = .038$, α_s: $F_1 = 5.05$, $P = .030$), but this difference was not observed when comparing their associations with submaximal VO_2 and the other self-reported measures (Ps $\geq .052$). Finally, the probability of transitioning from sedentary to active states (h_s) was more associated with submaximal VO_2 than with FACT-G total well-being ($F_1 = 4.12$, $P = .050$) and FACT-G physical well-being scores ($F_1 = 4.18$, $P = .048$), but this differential was not observed when comparing its associations with submaximal VO_2 and either FACT-G physical well-being 5-item subset scores ($F_1 = 2.55$, $P = .118$) or linked PROMIS-PF scores ($F_1 = 1.88$, $P = .179$).

For the remaining measures of activity fragmentation (the mean duration of sedentary bouts, μ_s, and the Gini index for sedentary bouts, g_s), we did not find that their association with submaximal VO_2 differed from those with FACT-G total well-being (Ps $\geq .139$), FACT-G physical well-being (Ps $\geq .071$), FACT-G physical well-being 5-item subscale (Ps $\geq .130$), or linked PROMIS-PF scores (Ps $\geq .160$).

Supplementary References

1. Brondeel R, Anaraki JR, Fuller D, KhataeiPour S. activityCounts: Generate ActiLife Counts. 2023.

2. Di J, Muschelli J, zipunnikov V. ActFrag: Activity Fragmentation Metrics Extracted from Minute Level Activity Data [Internet]. 2023. Available from: https://github.com/junruidi/ActFrag

3. Di J, Leroux A, Urbanek J, Varadhan R, Spira AP, Schrack J, et al. Patterns of sedentary and active time accumulation are associated with mortality in US adults: The NHANES study [Internet]. bioRxiv; 2017 [cited 2023 Mar 28]. p. 182337. Available from: https://www.biorxiv.org/content/10.1101/182337v1
